# Regeneration linked miRNA modify tumor phenotype and can enforce multi-lineage growth arrest in vivo

**DOI:** 10.1038/s41598-021-90009-9

**Published:** 2021-05-18

**Authors:** Siamak Salehi, Oliver D. Tavabie, Augusto Villanueva, Julie Watson, David Darling, Alberto Quaglia, Farzin Farzaneh, Varuna R. Aluvihare

**Affiliations:** 1 Institute of Liver Studies, King’s College Hospital, London, SE5 9RS UK; 2 MRC and Asthma UK Centre in Allergic Mechanisms of Asthma, King’s College London, Guy’s Hospital, London, UK; 3 School of Cancer & Pharmaceutical Sciences, King’s College London, Molecular Medicine Group, The Rayne Institute, 123 Coldharbour Lane, London, SE5 9NU UK

**Keywords:** Liver cancer, Cancer therapy

## Abstract

Regulated cell proliferation is an effector mechanism of regeneration, whilst dysregulated cell proliferation is a feature of cancer. We have previously identified microRNA (miRNA) that regulate successful and failed human liver regeneration. We hypothesized that these regulators may directly modify tumor behavior. Here we show that inhibition of miRNAs -503 and -23a, alone or in combination, enhances tumor proliferation in hepatocyte and non-hepatocyte derived cancers in vitro*,* driving more aggressive tumor behavior in vivo. Inhibition of miRNA-152 caused induction of DNMT1, site-specific methylation with associated changes in gene expression and in vitro and in vivo growth inhibition. Enforced changes in expression of two miRNA recapitulating changes observed in failed regeneration led to complete growth inhibition of multi-lineage cancers in vivo. Our results indicate that regulation of regeneration and tumor aggressiveness are concordant and that miRNA-based inhibitors of regeneration may constitute a novel treatment strategy for human cancers.

## Introduction

Although regenerative competence does not consistently correlate with organism complexity, in higher mammals this ability is restricted and the liver retains an unusual capacity amongst solid organs to regenerate^1^. Restricted regeneration in mammals has been partly attributed to the inability of fully differentiated, long-lived cells to enter cell cycle. Furthermore, robust cell cycle inhibitory pathways that control cancer development may restrict or promote regeneration in different settings^2,3^. In a number of animal models, ubiquitous cell cycle promoters and inhibitors have been shown to augment or block regeneration, respectively^4,5^.

We have previously identified specific and distinct miRNA species that appear to regulate successful and failed human liver regeneration^6^. In agreement with data from animal models, our results indicated that downregulation of a network of key miRNAs is important in determining regenerative outcome^7–9^. We hypothesised that this network of regeneration-linked miRNA may influence tumor biology and behaviour.

Here, we demonstrate that miRNA associated with successful regeneration drive more aggressive tumor phenotype and behavior. Conversely miRNA linked to failed regeneration can inhibit tumor growth, both in vitro and in vivo. Our data underscore the link between regulation of regeneration and tumorigenesis. Furthermore, they indicate a potential novel cancer treatment paradigm using inhibitors of regeneration.

## Results

**Figure 1:**
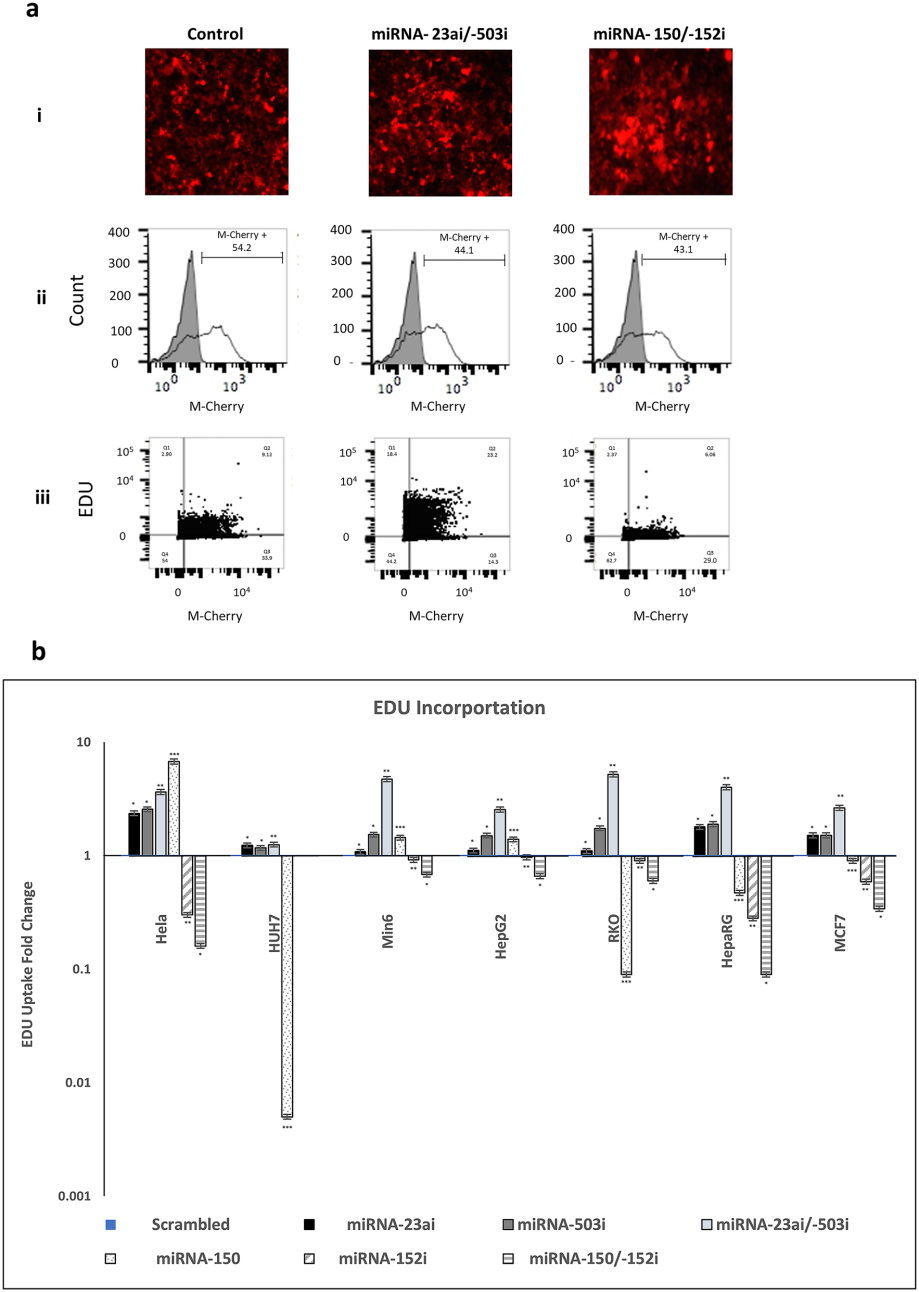
EDU incorporation. (**a**) Expression of miRNA-23ai/-503i and miRNA-150/-152i in HepG2 cancer cell lines under selection pressure. (i): Fluorescence images of samples transduced with scrambled, miRNA-23ai/-503i and miRNA-150/-152i. (ii): Flow cytometry profiles showing transduced HepG2 cells compared to un-transduced cells. All constructs co-expressed MCherry; bar represents the proportion of positively transduced cells. (iii): Flow cytometry dot plots showing EdU incorporation in HepG2 cells transfected with scrambled, miRNA-23ai/-503i and miRNA-150/-152i. (**b**) EdU uptake and fold change in Hela, HUH7, Min6, HepG2, RKO, HepaRG and MCF7 cell lines transduced with scrambled, miRNA-23ai, miRNA-503i, miRNA-23ai/-503i, miRNA-150, miRNA-152i and miRNA-150/-152i. The fold change of EDU + /M-Cherry + cells in each cell line are compared against scrambled control vector. Results are representative of three independent experiments. Error bars indicate SD. *p < 0.05,** p < 0.01, ***p < 0.001.

We investigated the influence of the miRNAs -503 and -23a, which we had observed to be downregulated during human liver regeneration, on tumor behavior in vitro. In parallel we investigated the effect of downregulation of miRNA -152 and upregulation of miRNA -150, which were associated with failed regeneration. Prior to the commencement of the study, we performed pathway analysis for the individual miRNA as well as co-expression of miRNA-503/-23a and miRNA-152/-150 (Supplementary Fig. 1). This confirmed that these miRNAs were involved with processes that govern regeneration. We transduced a model human liver cancer cell line HepG2 with lentiviral vectors interfering with the function of miRNA-503 (miRNA-503*i*), -23a (miRNA-23a*i*), -152 (miRNA-152*i*) or expressing -150 (miRNA-150). Homogeneous and high expressing transduced populations were obtained under selection, as confirmed by immunofluorescence and flow cytometry (Fig. 1ai and ii). Proliferation was assessed using 5-ethynyl-2′-deoxyuridine (EdU) incorporated during DNA synthesis and compared with proliferation in cells transduced with a scrambled control vector. In contrast to a proliferation index of 9% in cells transduced with control vector, cells expressing miRNA-503*i* and miRNA-23a*i* demonstrated a proliferation index of 23%, representing a greater than twofold increase in the number of proliferating cells over 5 h (Fig. 1aiii). In contrast, cells expressing miRNA-152*i* and miRNA-150 had a proliferation index of 6% over the same time period.

To investigate whether this phenomenon was cell type specific we investigated the effect of miRNA expression alone or in combination on proliferation in the following cell lines; HeLa (human cervical cancer cell line), HUH7 (human liver cancer cell line), Min6 (mouse pancreatic islet-cell derived cancer cell line), HepG2 (human liver cancer cell line), RKO (human colon cancer cell line), HepaRG (immortalized human liver cell line) and MCF (human breast cancer cell line) (Fig. 1b). Expression of miRNA-23a*i* or miRNA-503*i* alone consistently led to statistically significant increased proliferation in all cells, which was amplified by co-expression of these miRNA. Expression of miRNA-152*i* consistently led to reduced proliferation whilst miRNA-150 expression alone led to increased proliferation in Hela, Min6 and HepG2 cells lines. Co-expression of miRNA-152*i* and miRNA-150 however led to statistically significant and augmented inhibition of proliferation (when compared to miRNA-152*i* or miRNA-150 alone) in HeLa, Min6, HepG2, HepaRG and MCF cell lines.

**Figure 2:**
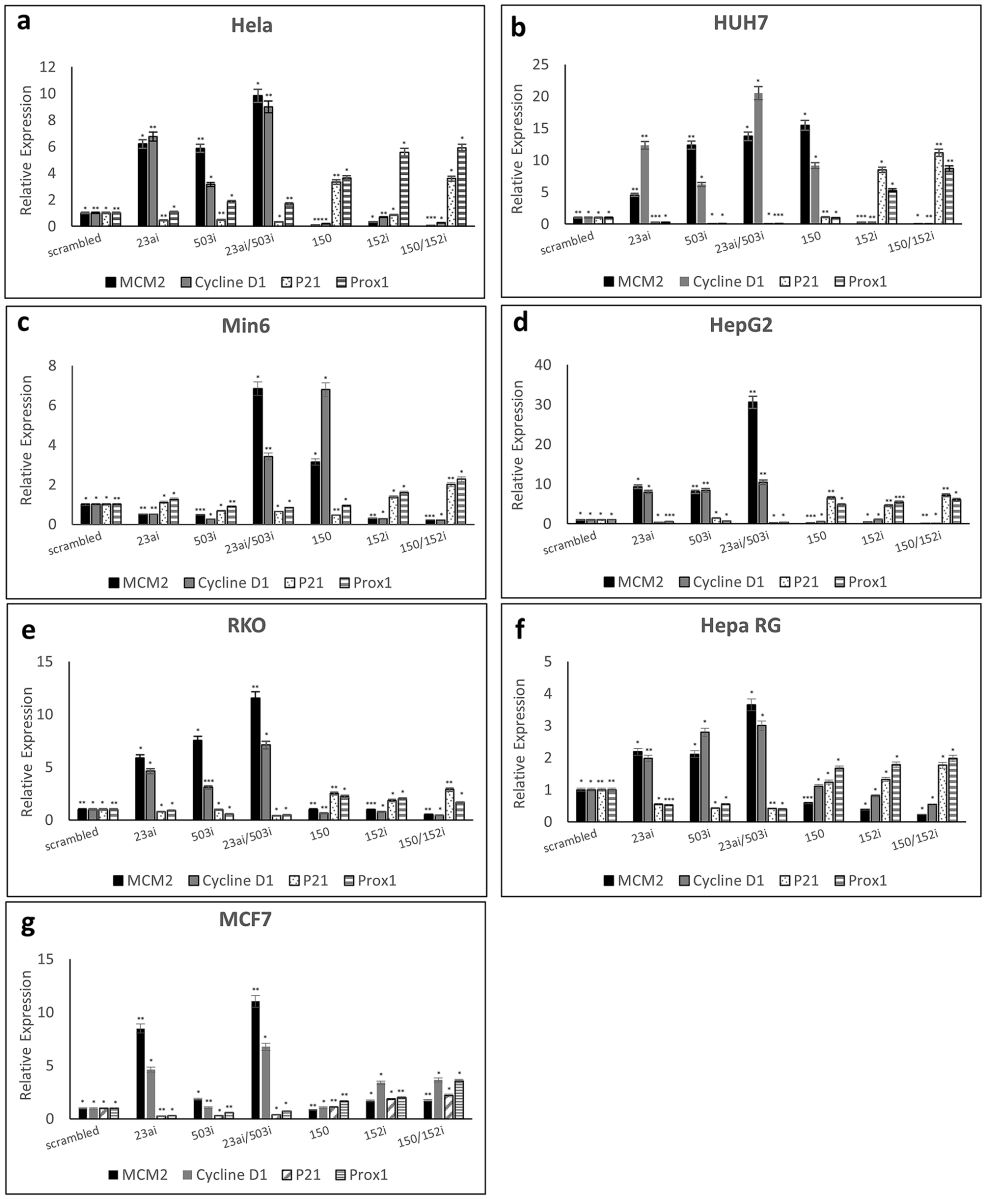
qPCR analysis of regulators of cell proliferation: MCM-2, Cyclin D1, p21 and Prox1. RNA templates were made from (**a**) Hela, (**b**) HUH7, (**c**) Min6, (**d**) HepG2, (**e**) RKO, (**f**) HepaRG and (**g**) MCF7 cells transfected with scrambled, miRNA-23i, miRNA-503i, miRNA-23ai/-503i, miRNA-150, miRNA-152i and miRNA-150/-152i. All expression levels were normalized to scrambled control vector. qPCR data in all cases are representative of three different experiments. Error bars indicate SD.

We next investigated whether the changes in cell proliferation we observed correlated with changes in expression of regulators of cell proliferation (Fig. 2a–h). When compared to cells expressing control vector, all cell-lines demonstrated up-regulated expression of minichromosome maintenance gene 2 (MCM2) and cyclin D1, both robust markers of cell cycle progression^10^, when co-expressing miRNA-23a*i* and miRNA-503*i*. With the exception of Min6, expression of miRNA-23ai or miRNA-503i was associated with up-regulation of MCM2 and cyclin D1 across cell-lines. In contrast, the expression of cell cycle inhibitors p21 and PROX1^11,12^ was not concordantly up- or down-regulated across cell lines. Expression of miRNA-152*i* alone in all cell lines led to consistently increased expression of the cell cycle inhibitors p21 and PROX1. Expression of miRNA-150 led to up-regulation of MCM2 and cyclin D1 in HUH7 and Min6 but up-regulation of p21 and PROX1 in HeLa, HepG2, RKO, HepaRG and MCF. Co-expression of miRNA-152*i* and miRNA-150 however led to up-regulation of p21 and PROX1 in all cell lines.

**Figure 3:**
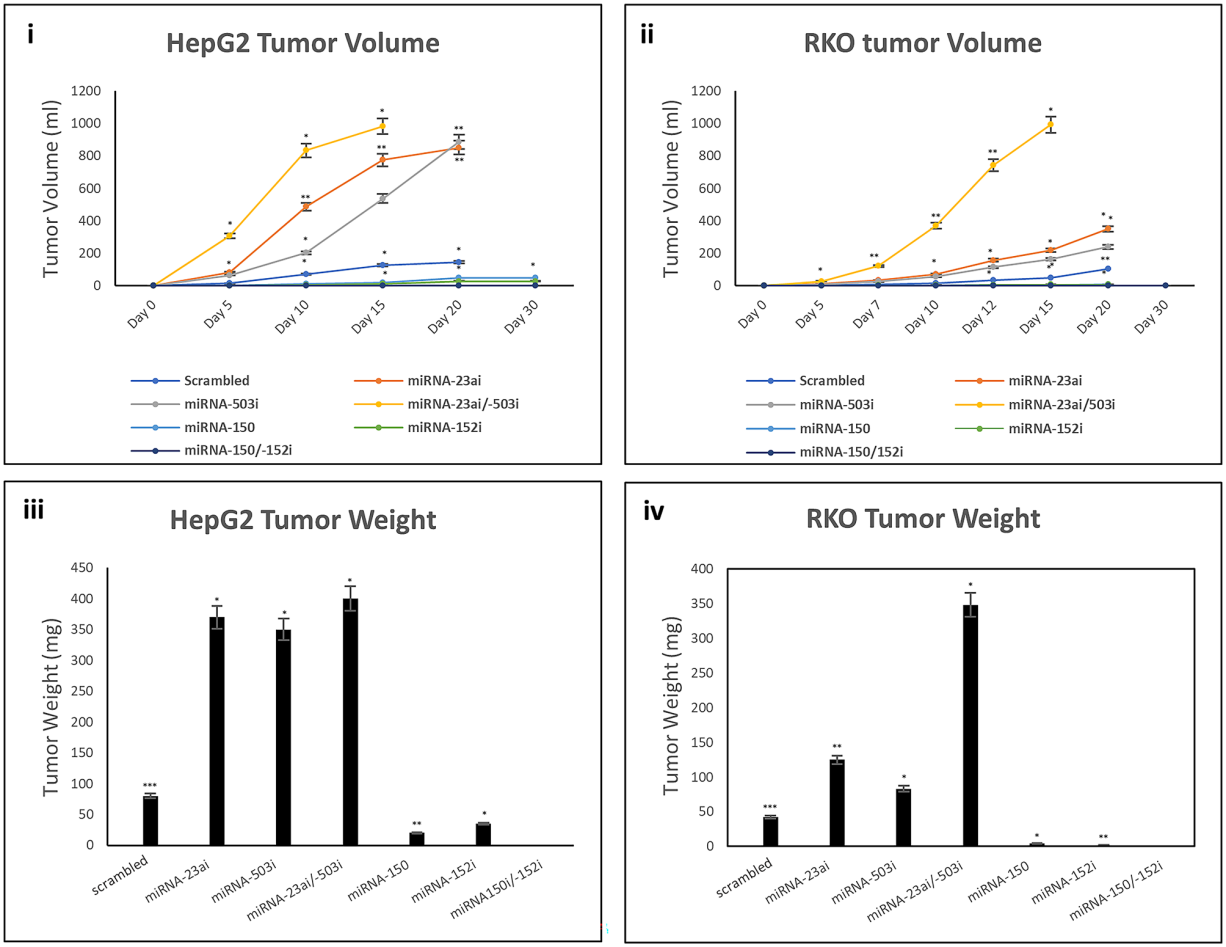
Growth kinetics. Tumor volume (ml) and weight (mg) measurement: tumor volume calculated from the recorded successive tumors in regular intervals using calipers to measure the longest and shortest tumor diameters (volume measurement formula explained in “Materials and methods”). Xenograft Tumor weights measured in nude mice at the end of experiments. (i): HepG2 Tumor Volume (ii): RKO tumor volume (iii): HepG2 tumor weight (iv): RKO tumor weight. Data is representative of three different experiments. *p ≤ 0.05; **p ≤ 0.01; ***p ≤ 0.001; p-values are relative to scrambled vector; SE = standard error.

We investigated whether the changes in proliferative rate we observed in vitro were associated with more aggressive tumor behavior in vivo, using a heterotopic xenograft model. Nude mice were injected in both flanks with pure populations of HepG2 or RKO cells expressing control vector, miRNA-503*i* or miRNA-23a*i* alone, or in combination. In vivo fluorescence imaging confirmed stable construct expression (Supp. Fig. 2a). In addition, we expressed miRNA-152*i* and miRNA-150 alone or in combination. These were assessed from the time of injection for growth kinetics, using tumor volume measurement and final tumor weight at the termination of the experiment (Day 20) (Fig. 3). We observed a statistically significant increase in the rate of growth between tumors expressing control vector and those expressing miRNA-503*i* or miRNA-23a*i* for both cell lines, which became more accentuated over time. The final tumor weight at Day 20 corroborated the volumetric data, showing a statistically significant difference between cells expressing control vector and those expressing miRNA-503*i* or miRNA-23a*i* (Fig. 3biii and iv). Co-expression of miRNA-503*i* or miRNA-23a*i* led to a statistically enhanced growth rate at all time points when compared to either construct alone in both cell lines, as measures by tumor volume or weight (3a).

**Figure 4:**
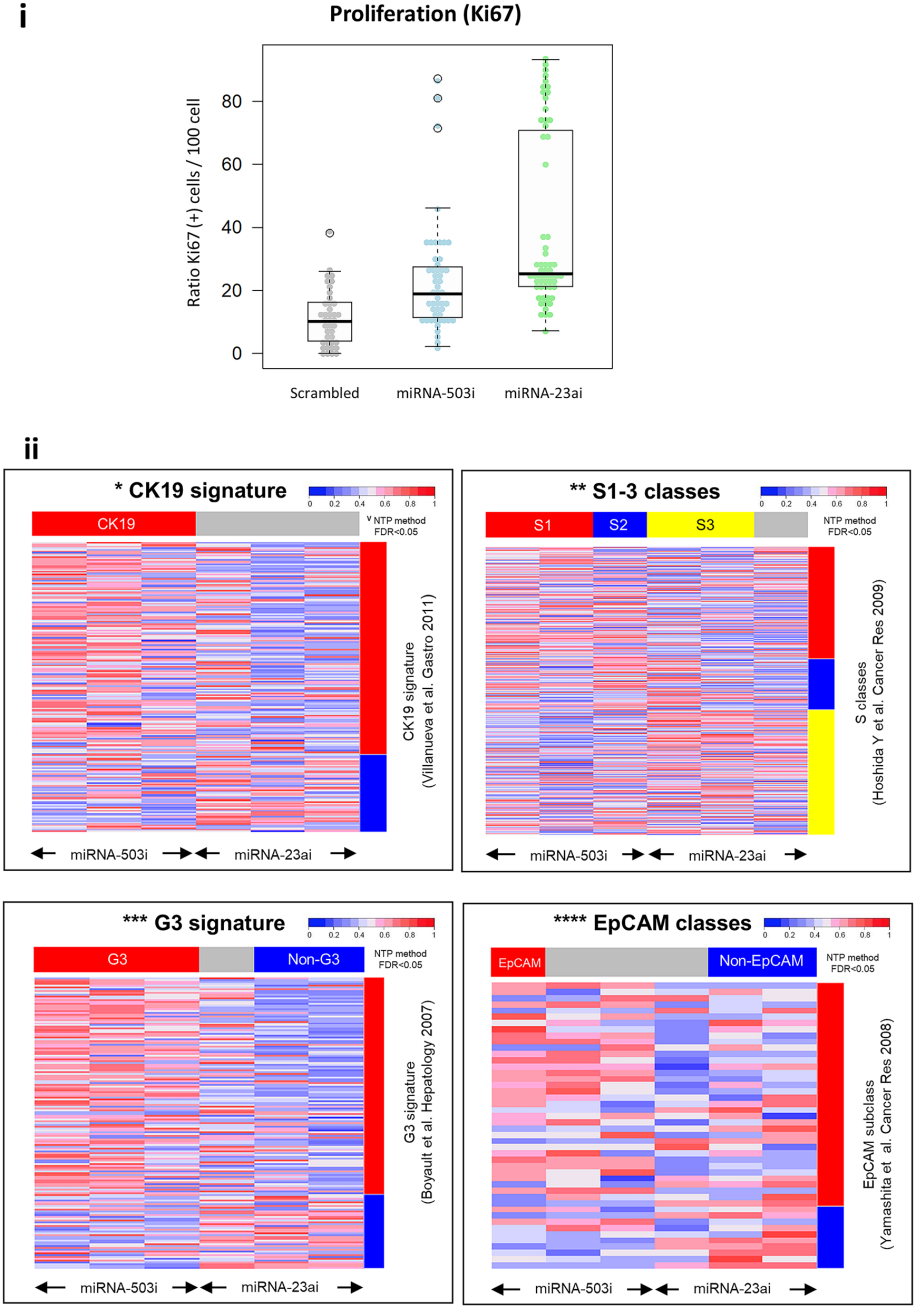
Ki67 proliferation index in xenograft tumors generated with miRNA constructs and comparison of cancer signature between these tumors and HCC cancer panels (i): Ki-67 expression ratio: Ki-67 positive cells per 100 cells obtained from tumor tissues transduced with scrambled control vector, miRNA-503i and miRNA-23ai. (ii): Comparing cancer signature between tumors developed in mice transduced with either miRNA-503i or miRNA-23ai using NTP method with FDR < 0.05 looking at known indicators of HCC. *CK-19: (cytokeratin-19). **S1–S3: (three robust HCC subclasses (termed S1, S2, and S3), each correlated with clinical parameters such as tumor size, extent of cellular differentiation, and serum alpha-fetoprotein levels. An analysis of the components of the signatures indicated that S1 reflected aberrant activation of the WNT signaling pathway, S2 was characterized by proliferation as well as MYC and AKT activation, and S3 was associated with hepatocyte differentiation. ***EpCAM: (epithelial cellular adhesion molecule). ****G3: (Glypican 3). VNTP method: Nearest-Template Prediction method (NTP, extensively reviewed, Hoshida Y. 2010).

On termination of these experiments the tumors were explanted and analyzed by histology and immunohistochemistry. Ki-67 immunostaining and quantification of the proliferative index again confirmed significantly increased proliferation in tumors expressing miRNA-503*i* or miRNA-23a*i* when compared to those expressing the control vector (*P* < 0.001) (Fig. 4). Comparison of the gene expression signature obtained from explanted xenograft tumors expressing miRNA-503*i* or miRNA-23a*i* with known human clinically validated prognostic signatures revealed that expression of either miRNA adversely altered the gene expression into a poor prognostic category (Fig. 4).

Conversely, expression of miRNA-152*i* and miRNA-150 alone in both HepG2 and RKO cell lines led to statistically significant lower tumor volume in vivo compared to those expressing the control vector (Fig. 3a). These differences were also accentuated over time and corroborated with reduced tumor weight in both cell lines expressing miRNA-152i and miRNA-150. Co-expression of both constructs led to complete growth arrest for 30 days as measured by tumor volume and final weight.

**Figure 5:**
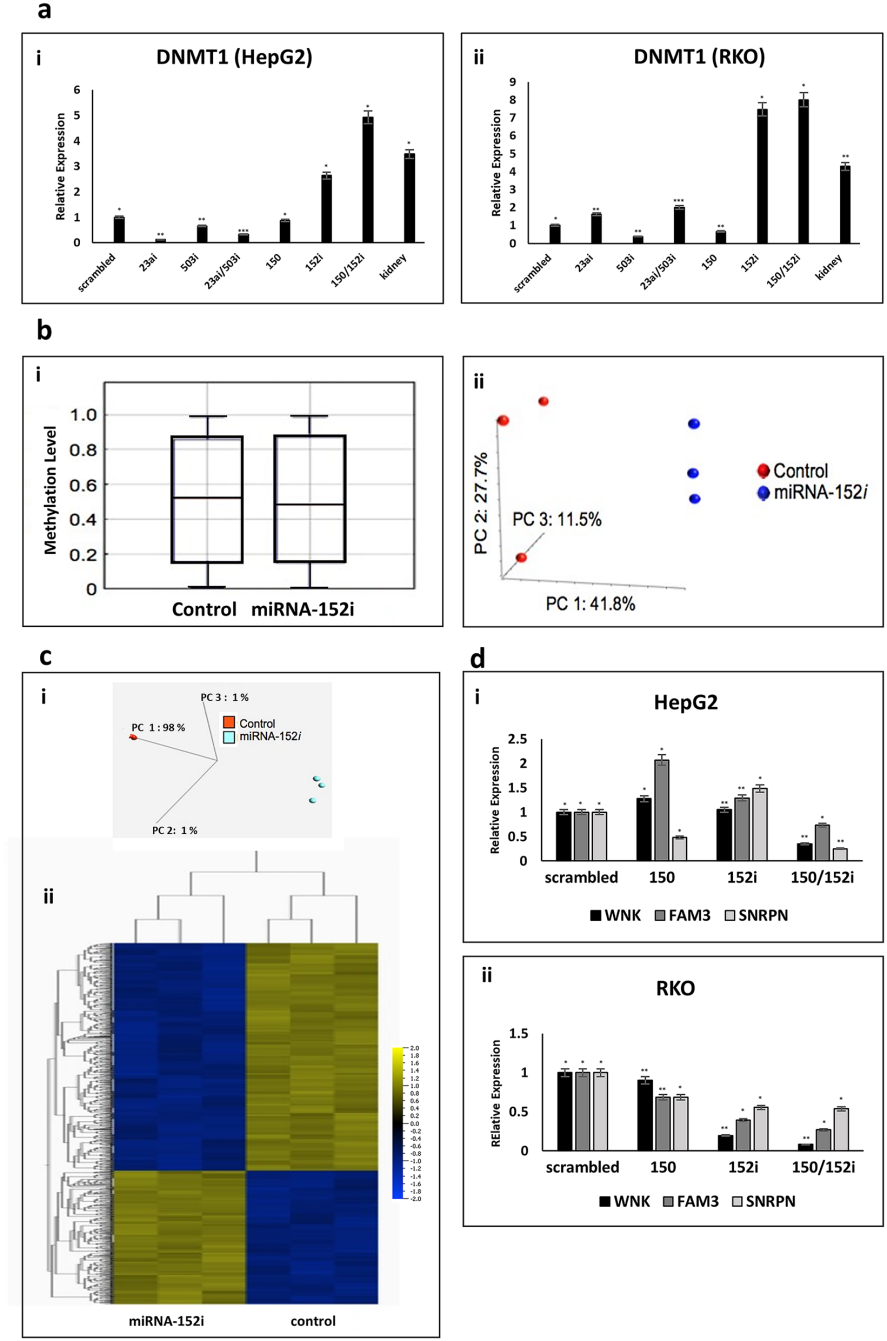
DNMT1 expression in HepG2 and RKO cells transfected with miRNA constructs, methylation and gene expression arrays in miRNA-152i generated xenograft tumors and expression of genes associated with cell proliferation and cancer in HepG2 and RKO cells transfected with miRNA constructs. (**a**) qPCR analysis of DNMT1 expression. (i) HepG2 and (ii) RKO cells transfected with miRNA constructs. Kidney RNA used as a positive control. All expression levels were normalized to scrambled control vector. qPCR data in all cases are representative of three different experiments. Error bars indicate SD. *p < 0.05, **p  < 0.01, ***p < 0.001. (**b**) Analysis of the total CpG methylation in cells transduced with scrambled control vector and miRNA-152i. The datasets are equivalent, overlapping and not skewed (3% difference); (i): box and whisker plot, (ii) principle component analysis (PCA) of the degree of methylation at each CpG in the two datasets shows samples group by condition. (**c**) Gene expression analysis using Affymetrix gene2 array: RNA extracted from xenograft tumors transduced with scrambled or miRNA-152i. (i) PCA shows complete segregation of 2 groups indicating differentially expressed genes within each group, (ii)The dendrogram shown above heatmap demonstrates similarities and differences between samples by miRNA expression (Affymetrix GeneChipm Command Console (AGCC) 4.0, http://www.affymetrix.com/support/technical/byproduct.affx?product=commandconsole). (**d**) qPCR analysis of SNRPN, WNK3, and FAM3B genes. (i) HepG2 and (ii) RKO) cells transfected with scrambled, miRNA-150, miRNA-152i and miRNA-150/-152i. All expression levels were normalized to scrambled control vector. qPCR data in all cases are representative of three different experiments. Error bars indicate SD. *p < 0.05, **p < 0.01, ***p < 0.001.

To further investigate the growth arrest observed in cell lines expressing miRNA-152i and miRNA-150, we evaluated the expression of DNA methyltransferase 1 (DNMT1) in both cell lines. Down regulation of miRNA-152, through regulation of DNMT1, is known to cause hypermethylation, aberrant gene expression and cell cycle inhibition^13,14^. HepG2 and RKO cells expressing miRNA-152*i* demonstrated statistically significant high-level gene expression of the key target gene DNMT1, which was augmented by co-expression of miRNA-150 in both cell lines (Fig. 5a). We next investigated whether the miRNA-152*i*-driven changes in DNMT1 expression we observed altered DNA methylation using the Illumina Infinium Human Methylation 450K arrays. Analysis of global methylation revealed that mean methylation at all CpG sites for tumors expressing control vector was 52%, compared to 48.5% for tumors expressing miRNA-152*i* (Fig. 5bi). However, analysis revealed site-specific increased methylation in cells expressing miRNA-152*i* (Supp. Fig. 3). PCA analysis confirmed that tumors expressing miRNA-152*i* had distinct methylation profiles when compared with tumors expressing control vector (Fig. 5bii).

We carried out gene expression analysis comparing the control xenograft tumors with those expressing miRNA-152*i,* to assess whether in vivo expression of miRNA-152*i* specifically altered global gene expression. We demonstrated significant differential gene expression by PCA and cluster analysis (Fig. 5c). We therefore investigated whether the miRNA-152*i* induced site-specific hypermethylation identified in vitro could be correlated with specific changes in gene expression from the same cancers in vivo. We identified three genes, Small nuclear ribonucleoprotein polypeptide N (SNRPN), WNK3 and Family with sequence similarity 3B (FAM3B) that demonstrated concordant CpG methylation and significantly reduced expression by gene expression array. We confirmed specific and significant reduced expression of these 3 genes in both HepG2 and RKO cells due to expression of miRNA-152*i* by quantitative PCR (Fig. 5d).

## Discussion

Our findings indicate that key miRNA regulators of human liver regeneration can alter tumor behavior in liver and non-liver derived cancer cell lines. Whilst further mechanistic investigation is required, these observations raise the possibility that regenerative drive confers a more generalized, rather than tissue–specific, cancer risk. Although our data pertain to hepatocyte-derived tumors, it is noteworthy that regeneration-linked stem cell proliferative drive correlates with cancer propensity^15^. miRNA regulators of liver regeneration induced large scale gene expression changes that regulate biological pathways associated with tumor behavior and also enhanced tumor aggressiveness in vivo. In addition, our findings demonstrate that inhibition of miRNA-152 can inhibit tumor growth in vitro and in vivo. Previous reports have demonstrated an association between downregulation of miRNA-148/152 expression and some gastrointestinal cancers^16,17^. The contrast with our findings may reflect the specific dosage effect of miRNA-152 expression in our experiments or the impact of cell lineage. The tumor inhibitory capacity of miRNA-152 was linked to site-specific methylation of target genes, likely through increased expression of DNMT1 although further investigation is required to explore the effect of miRNA-152 across potential candidate genes. We observed methylation-induced changes in expression of three genes associated with cell proliferation and cancer. The WNK family of kinases have been associated with cell cycle progression, metastasis and metabolic adaptation in tumor cells^18,19^. The FAM3 family have been associated with hepatic metabolic regulation and tumor formation/metastasis^20,21^. SNRPN is linked to Prader-Willi syndrome and is involved in pre-RNA processing and splicing^22,23^. Targeted inhibition of expression SNRPN, WNK3 and FAM3B may therefore constitute a potential therapeutic strategy for HCC and more generally for other cancers. Our finding that co-expression of miRNA augmented the tumor modulatory effect of pro- or anti-regenerative miRNA is compatible with our previous observation of concerted, rather than individual changes in expression of miRNA during regeneration. The more pleotropic inhibitory activity of the co-expression miRNA-152*i* and miRNA-150 in non-hepatic lineage cancer cells known to demonstrate more aggressive tumor biology, indicates that this combination may have a more general anti-cancer applicability, subject to further evaluation.

Given the concordance of regenerative regulation and tumor behavior, our findings may help to explain in part, why regenerative capacity is so restricted in higher eukaryotes. Furthermore, they may explain the high incidence of cancers that arise in the liver, representing a global health burden, which may result from preserved regenerative competence^24^. Clinical data indicate potentially deleterious effects of liver regeneration, induced by therapeutic interventions such as live-donor liver transplantation and liver resection, on tumor biology and outcomes in the context of primary liver cancer^25^ and metastatic tumors^26^. Our findings may provide a mechanistic basis of these clinical observations.

Cancers can also arise in other organs with poor regenerative capacity due to faciliatory changes in tumorigenic- or tumor suppressor-pathways^27^. The possibility that they may also do so either by subverting dormant regenerative pathways in terminally differentiated cells or by inducing stem cell proliferation^12^, remains to be tested. Finally, our findings highlight a potentially novel avenue of anti-cancer therapy, by targeting miRNA regulators that inhibit regeneration. These agents have the potential not only to prevent tumor growth in vivo, but may also alter tumor responsiveness to existing treatment modalities and require further investigation.

## Materials and methods

### Study approval

All experiments were approved by the ethics committee at King’s College London and follow approved King’s College London protocols. All animal work in this study is performed in accordance with the Animals (Scientific Procedures) Act 1986 (ASPA) and amendment regulations 2012 covered by UK Home Office Project license PPl 70/7834 and King’s College London establishment license number X24D82DFF. This study was carried out in compliance with the ARRIVE guidelines.

### In vivo animal experiments

#### Cell lines

Hela, HepG2, RKO and MCF-7 were purchased from American Type Culture Collection, USA. HepaRG cells were purchased from Sigma-Aldrich, Germany. HUH and Min6 cells were gifted from Haematological Medicine Department, King’ College London.

All cell culture materials were purchased from Gibco-BRL, UK. Cells were cultured and preserved in conditioned growth medium according to company protocols^28^.

### Pathogen testing

All cell lines were independently tested against all relevant mouse pathogens, including mycoplasma, prior to in vivo experiments (Mouse Essential PCR panel, Charles River Laboratories Inc., USA), and were certified as being pathogen-free.

### Animals

Six-to-eight week-old female BALB/c nude mice (20–25 g; Harlan animals) were selected as recipients for the transduced cells. The mice were maintained in filter cages under specific pathogen-free conditions in the Comparative Biology Centre at King’s College London, in accordance with the Home Office guidelines for Animal Scientific Procedures UK.

### Tumor formation assay

Huh7 cells were transduced with lentivirus-expressing inhibitors of the miRNA of interest or a scrambled control vector. Cells were harvested and resuspended in PBS to a final concentration of 50 × 106 cells/ml. Xenograft tumors were generated by subcutaneous injection of 5 × 106 Huh7 cells suspended in 100 μl PBS into both lower flanks of the mice, using a 25-gauge needle. The cells were kept on ice during the time between harvest and injection. Three mice were injected for each construct and one mouse was kept as a control without any injection. All animal experiments were conducted in triplicates and each experiment was repeated three times.

### Tumor volume measurement

Tumor volumes were calculated from the formed tumors using calipers to measure the longest and shortest tumor diameter. Tumor volume was calculated as: Volume = da × db^2^, with da and db being the longest and shortest tumor diameters, respectively. Tumors were measured and recorded twice a week. No tumor was allowed to progress beyond 15 mm in diameter. On Day 21 or when a tumor had reached a maximum volume of 1000 ml or a maximum diameter of 15 mm (if less than 1000 ml), the animals were sacrificed by schedule 1 method. An incision was made in the skin over the subcutaneous tumor and the tumor tissue was removed by blunt dissection. Tumor weight was measured using digital scales. A total of 200 ml of blood was taken by cardiac puncture for biochemistry analysis.

### BRDU assay

To determine the viability and proliferative status of the infected areas in the tumors, mice were injected with 1 ml of concentrated BrdU per 100 mg body weight (Invitrogen, UK; cat no: 00-0103) on Day 21. After 2 h animals were sacrificed in order to determine the number of DNA replicating cells in the Huh7 tumors. DNA-incorporated BrdU was detected by a three-step immunoperoxidase staining with the anti -BrdU monoclonal antibody.

### Sample processing for human gene 2.0 ST arrays

Total RNA was extracted using Trizol reagent (Invitrogen, UK) from xenograft tumor tissues and quality and integrity were assessed using ribosomal RNA band analysis on a 2100 Bioanalyser and RNA 6000 Nano LabChips (Agilent, UK). 75 ng of total RNA was reverse transcribed and amplified into cDNA using NuGEN’s Pico WTOvation labeling kit, following the manufacturer’s protocols (NuGEN Inc, CA, USA). The Exon conversion module (NuGEN Inc, CA, USA) was used to synthesize a sense orientation copy from the amplified cDNA, and the Biotin module for biotin-labelling. NuGEN’s recommendations were followed for hybridization to Affymetrix Human Gene 2.0 ST arrays (Affymetrix, CA, USA) and subsequent processing using standard hybridization, washing and staining reagents (Hybridisation Wash Stain (HWS) kit. Scanned array images (DAT and CEL files) were generated using Affymetrix’s AGCC software, and analyzed using their Expression Console package, which generates normalized, background-corrected probeset-summarized signals for each gene on the array. The standard gene-level RMA workflow was used to achieve this data output. Control probeset data were removed from the main dataset prior to data analysis, through the deletion of rows containing information for various ‘normgene’ probesets.

### Statistics

The filtered data table was formatted as a ‘.gedata’ tab-delimited text file and imported into Qlucore’s Omics Explorer 2.1 software for analysis. The software, which utilizes a visual, Principal Components Analysis (PCA) approach to display the relationships between samples and genes, allowed the selection of differentially expressed genes using standard statistical techniques. A simple *t* test was employed to select genes that were differentially regulated across the different sample groups (xenograft tumors in nude mice injected with cells transduced with inhibitors of miRNAs -152 or scrambled control vector), using the P value slide bar to create the various statistical cut-off gene lists for the different comparisons of interest. Gene lists (containing all regulated genes) were displayed as heat maps to show gene expression patterns within the list, and sub-lists of interest were selected on the basis of specific expression patterns.

### Metacore pathway analysis

The Analyze Networks algorithm was used to demonstrate biological processes associated with miRNA signatures developed. This uses a library of greater than 24,000 human genes in networks to predict pathways likely to be associated with inputted data. The algorithm generates and ranks biological networks based on:- (1) the relative enrichment of the network with the uploaded data (miRNA) and (2) the relative saturation of these networks with canonical pathways.

These networks are built on the fly and unique for the uploaded data. In this workflow the networks are prioritized based on the number of fragments of canonical pathways on the network and then Enrichment analysis used for matching gene IDs of possible targets for the "common", "similar" and "unique" sets with gene IDs in functional ontologies in MetaCore. The probability of a random intersection between a set of IDs the size of target list with ontology entities is estimated in p value of hypergeometric intersection.

### HIV-derived disabled lentiviral miRNA expression vectors

Lentiviral miArrest inhibitor vectors (pEZX-AM04, Genecopoeia, USA) for miRNAs -152, -503 and -23a and miRNA expression clones (pEZX-MR03 Genecopoeia, USA) contained a GFP reporter gene, a puromycin selectable marker and a U6 promoter. miRNA inhibitor constructs bind specifically to their target miRNA to block miRNA gene regulation, resulting in the up-regulation of specific miRNA target genes. The miRNA inhibitor scrambled control clone for pEZXA-M04 expressing mCherry and puromycin were used as a control.

### HIV lentiviral vector production and processing

Transient transfection of BL15 cells produced VSV-G pseudotypes after transfection with pCMV∆R8.91, pMD.G and the pEZX-AM04 miArrest inhibitor vector with a ratio of 7:3.5:9.5 μg, respectively, using calcium phosphate co-precipitation. After 24 h BL15 cells were washed four times in serum-free DMEM and cultured for 24 h in serum free DMEM. The vectors were calcium phosphate concentrated as described previously, and 47 ml of lentivirus was reduced to a pellet to which 900 μl of modified solubilisation buffer was added (100 mM EDTA, 50 mM NaCl, 0.2% BSA, pH 6.5), giving a final volume of 1.1 ml. The final solution was added to 5 × 108 pelleted cells, prewashed (2 × 400 μl HBSS + 0.1% BSA) streptavidin superparamagnetic particles (Promega, USA) and incubated for 18 h under constant agitation. Particles were then washed and resuspended in HBSS + 0.1% BSA. The concentrated preparations were then used for infecting their target cell lines.

### Fluorescence-activated cell sorting (FACS)

72 h post infection, transduced cells were sorted into positively- and negatively-transduced populations using a BD FACS Aria Cell Sorter. mCherry expression was detected with the Yellow/Green (560 nm) laser, 600 nm long pass mirror and 610/20 filter. Pacific blue expression was detected with the Violet (405 nm) laser, 450/50 filter. Flow cytometry data were analyzed using FlowJo version 7.6.5.

### Analysis of puromycin expression by RT-PCR

RT-PCR was performed using the PCR Enzyme Selection Kit (Invitrogen, UK). Primers were purchased from Eurofins MWG Operon. Forward sequence: ttcgccgactacccc; reverse sequence: tagaaggggaggttgc.

### Gene expression analysis by qPCR

MCM2, p21, cyclin D1 and PROX1 expression were analyzed using TaqMan Gene Expression Assays (Applied Biosystems, USA). Expression levels were normalized to a scrambled control vector and data were expressed as mean ± standard deviation. The Student’s t-test was used to determine significance. P values < 0.05 were considered significant.

DNMT1 expression was analyzed using TaqMan Gene Expression Assays (Applied Biosystems, USA) and expression levels were normalized to a control cDNA obtained from liver tissue. cDNA from kidney tissue served as a positive control. FAM3B, SNRPN and WNK3 expression were also analyzed using TaqMan Gene Expression Assays (Applied Biosystems, USA). Expression levels were normalized to a scrambled control vector.

### EdU cell proliferation assay

Huh7 and HepG2 cells (human hepatocyte derived cellular carcinoma cell lines) were maintained in MEM supplemented with 10% heat inactivated fetal bovine serum and an antibiotic/antifungal solution. MIN6 cells (pancreatic β cell line derived from a mouse insulinoma) were maintained in RPMI medium with the same supplements. Cell cultures were maintained at 37 °C under 5% CO_2_. All cell culture materials were purchased from Gibco-BRL, UK.

Cell proliferation was analyzed using the Click-iT EdU Pacific Blue Flow Cytometry Assay Kit (Invitrogen, UK). Huh7, HepG2 and MIN6 cells were seeded in six-well plates and infected with 50 μl (MOI of 10) of miRNA/puromycin-expressing lentiviral vectors. Cells were cultured at 37 °C, 5% CO_2_ and the cell culture media was replaced after 24 h with medium containing 4 μg/ml puromycin. After 3 days, all uninfected cells had died generating colonies of stable cells in culture. Puromycin selection pressure was maintained for another week with daily fresh medium containing puromycin. After 1 week, colonies were selected using an inverted fluorescence microscope with a Gilson pipette and yellow tip. Colonies were expanded by transferring to a 24-well plate containing maintenance dose of puromycin (1 μg/ml). 90–95% confluent cells were transferred into a single well of a six-well plate and kept in maintenance dose of puromycin. Cells were maintained in culture for another four days, after which they were treated with 10 μM EdU for 5 h. EdU incorporation was detected according to the manufacturer’s instructions.

### Immunohistochemistry

Tissue samples were formalin fixed and paraffin embedded. The cell morphology was initially assessed with hematoxylin and eosin stain. A further assessment of morphological and functional phenotype was performed by immunohistochemistry using antibodies against human hepatocyte antigen (Hep Par-1; Dako, dilution 1:200), Glypican 3, Sigma, dilution 1:100, BrdU, Abcam dilution 1:100 and Ki-67, Dako, dilution 1:25). The immunostains were carried out using an automatic immunostainer (Bond Max, Leica Microsystems, Wetzlar, Germany), including the nuclear counter-staining. Sections were mounted in DPX mountant. The sections were examined by a liver histopathologist (AQ) who was blind to the status of each sample in terms of xenograft injection. Tumor cells, mitotic figures and nuclei staining for Ki-67 and BrdU were counted using a Glasgow cell counting graticule, Datasights Limited. For each sample, 10 fields were randomly selected at 400× magnification for the mitotic count. Each field was marked and the ki-67 and BrdU count was repeated in the same area in the sections used for immunohistochemistry. Glypican and Hep Par-1 expression were assessed using a semi-quantitative scale , as follows: 0 = No staining; 1 = Very focal staining observed after careful examination at high magnification; 2 = focal staining spotted at low magnification; 3 = staining easily spotted at low magnification, but minority of cells tumor cells staining; 4 = majority of tumor cells staining; 5 = virtually all tumor cells staining with no negative tumor cells present.

### Methylation array

Genomic DNA extraction DNA was extracted using Trizol reagent (Invitrogen, UK) and DNA properties were assessed by agarose gel electrophoresis and NanoDrop 2000c Spectrophotometer (Thermo Scientific, USA).

Bisulphite conversion of genomic DNA and Genome-wide methylation analysis 500 ng of genomic DNA was treated with sodium bisulphite using Zymo EZ DNA methylation kit (Zymo Research Irvine, USA) and tested for conversion by PCR using primers specific for bisulphite modified DNA. Genome-wide DNA methylation was assayed using the Illumina Infinium Human Methylation 450K beadchip and raw data signals were obtained using GenomeSudio software. Data were exported for further analysis using Partek Genomics Suite.

## Supplementary Information

Supplementary Information.
