# Supplementary material for: Regeneration linked miRNA modify tumor phenotype and can enforce multi-lineage growth arrest in vivo
Source: Sci Rep. 2021 May 18;11:10538. doi: 10.1038/s41598-021-90009-9 (PMC8131690; doi:10.1038/s41598-021-90009-9)
Supplement: Supplementary file 1 — Supplementary Information. [file 41598_2021_90009_MOESM1_ESM.pdf]

Regeneration linked miRNA modify tumor phenotype and can enforce multi-lineage growth arrest in vivo

Siamak Salehi1, Oliver D Tavabie1, Augusto Villanueva1, Julie Watson2, David Darling3, Alberto Quaglia1, Farzin Farzaneh3 and Varuna R Aluvihare1*

a

i

miRNA-23A	P-value
Adipocyte Differentiation	0.0231
Antiviral Immunity	1.13E-03
Apoptosis	0.0597
Cell Death	0.0439
Cellular Senescence	3.94E-03
DNA Damage Response	9.00E-03
Dendritic Cell Differentiation	2.25E-03
Glucose Metabolism	0.0158
Hormone-mediated Signalling Pathway	0.0326
Mesenchymal Stem Cell Proliferation	4.50E-03
Mesenchymal-to-Epithelial Transition	2.25E-03
Muscle Development	0.0118
Natural Killer Cell Activation	2.81E-03
Neuron Apoptosis	8.44E-03
Wound Healing	0.0129

iii

miRNA-150	P-value
Angiogenesis	0.0366
Apoptosis	0.0597
Cell Cycle	0.0467
Cell Differentiation	0.0315
Cell Proliferation	0.045
Cytokine Secretion	1.13E-03
Haematopoiesis	0.0321
Immune Response	0.0518
Immune System	0.0118
Inflammation	0.063
Onco-MiRNAs	0.0208
T-Cell Differentiation	9.00E-03
Toxicity	0.0203

ii

mi-RNA-503	P-value
Cell Cycle	0.0467
Cell Differentiation	0.0315
Cell Proliferation	0.045
Osteoclast Differentiation	7.88E-03
Oxidative Stress	5.63E-03

iv

mi-RNA-152	P-value
Cell Migration	6.19E-03
Embryonic Development	9.57E-03
Endothelial Cell Proliferation	3.83E-03
Peritoneal Cavity Homeostasis	0.0129
T-helper 17 Cell Differentiation	0.0107

b

i

Biological Networks miRNA-150/miRNA-152	
-Regulation of cell differentiation<br>-Negative regulation of macrophage derived foam cell differentiation<br>-Negative regulation of cholesterol storage<br>-Negative regulation of lipid localization	6.95E- 04

ii

Biological Networks miRNA-23a/miRNA-503	P-value
-Positive regulation of cell cycle<br>-Regulation of cell cycle phase transition<br>-Positive regulation of mitotic cell cycle phase transition<br>-Regulation of mitotic cell cycle phase transition<br>-positive regulation of cell cycle phase transition	5.30E-07
-Tissue development<br>-Positive regulation of cell differentiation<br>-Regulation of cell differentiation<br>-Positive regulation of transcription by RNA polymerase II	2.58E-08

Supp. Fig 1: Individual miRNA Functions and biological networks associated with paired miRNA

a: miRNAs were individually uploaded to investigate their functional associations, the software can “Analyze” enrichment of uploaded miRNAs in the curated miRNA sets, by using the statistical overrepresentation analysis. i: Functions associated with miRNA-23a, ii: Functions associated with miRNA-503, iii: Functions associated with miRNA-150 and iv: Functions associated with miRNA-152

b: MetaCore pathway analysis was performed on i: miRNA-150 and -152 and ii: miRNA-23a and -503 to identify biological networks associated with paired miRNA. Further explanation of methods utilised are in the statistical methods.

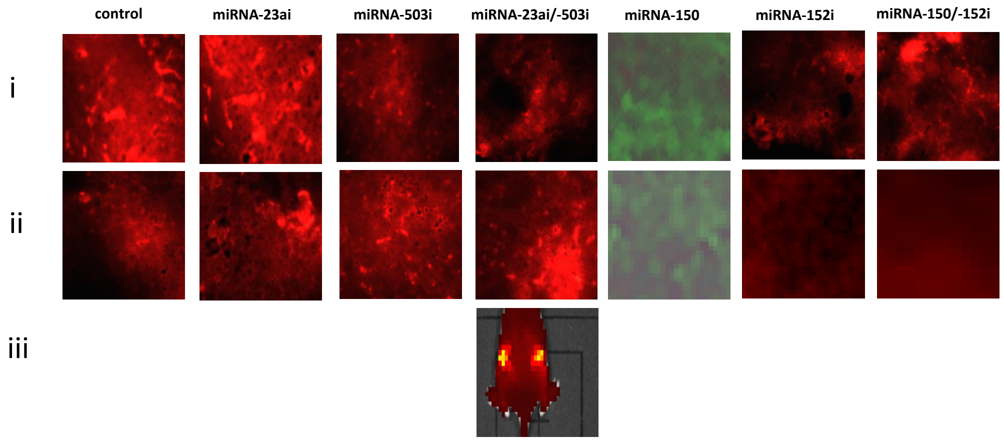

Supp. Fig. 2 : Xenograft tumors

Tumors generated by injecting 5x10^6 pure population of transfected cells expressing scrambled vector as control, miRNA-23ai, miRNA-503i, miRNA-23ai/503i, miRNA-150, miRNA-152i and miRNA-150/-152i in nude mice flanks. Tissue imaging using florescence microscope showing M-Cherry and GFP expression in (i): tumors generated with HepG2 transfected cells and (ii): tumors generated with RKO transfected cells. (iii): in-vivo imaging using IVIS in-vivo imaging machine showing M-Cherry expression in tumors generated with RKO cells transfected with miRNA- 23ai/-503i.

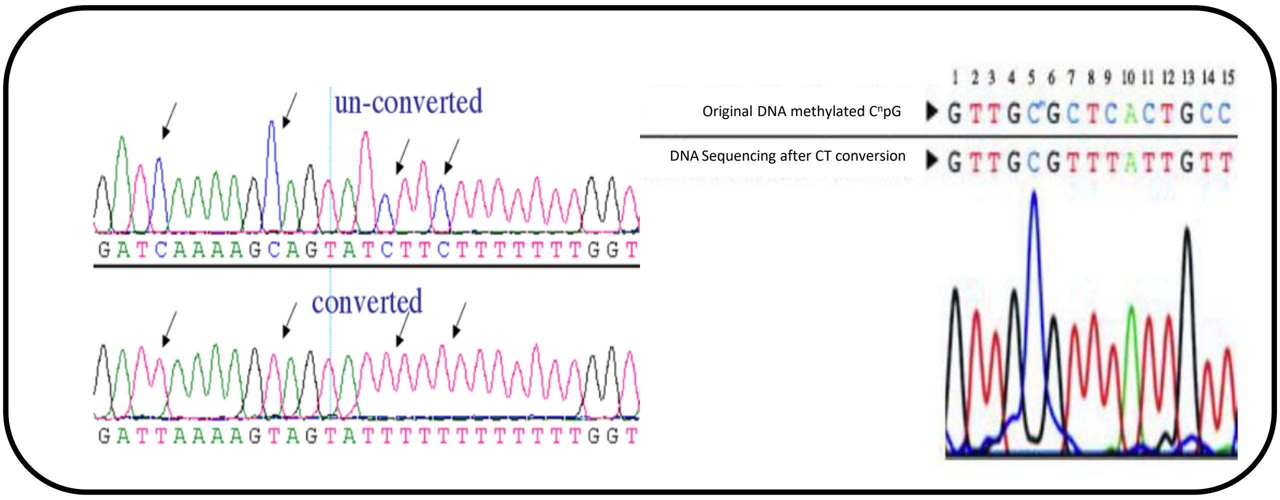

Supp. Fig. 3: Methylation status of cells transduced with miRNA 152 inhibitor using bisulfite sequencing. Genomic DNA was treated with sodium bisulphite to identify methylated CpG islands from non-methylated CpG islands in cells transduced with miRNA-152 inhibitor. The conversion was checked by methylation specific PCR and sequencing using primers specific for bisulphite modified DNA. For further information, see material and methods
